# Split‐Window OCT biometry in pseudophakic eyes

**DOI:** 10.1111/aos.15198

**Published:** 2022-06-07

**Authors:** Bartosz L. Sikorski, Kenneth J. Hoffer

**Affiliations:** ^1^ Department of Ophthalmology Nicolaus Copernicus University Toruń Poland; ^2^ Clinical Professor of Ophthalmology University of California, Los Angeles Stein Eye Institute Los Angeles California USA; ^3^ St. Mary's Eye Center Santa Monica California USA

**Keywords:** biometry, OCT, pseudophakic eyes, Split‐Window OCT, SW‐OCT

## Abstract

**Purpose:**

To determine the utility of Split‐Window optical coherence tomography OCT (SW‐OCT) biometry in measuring ocular axial dimensions as well as imaging the intraocular lens (IOL) and posterior capsule in pseudophakic eyes.

**Methods:**

Sixty‐nine pseudophakic eyes of 69 subjects were enrolled in the study. The results of SW‐OCT biometry implemented in the SD OCT device for posterior and anterior segment imaging (REVO NX, Optopol Technology) were compared with those obtained with the SS‐OCT‐based biometer IOLMaster 700 (Carl Zeiss Meditec). Differences in measurement values between the two biometers were determined using the paired *t*‐test. Agreement was assessed through intraclass correlation coefficients (ICC) and Bland–Altman plots.

**Results:**

The correlation between measurements obtained with SW‐OCT and SS‐OCT was very high (ICC for: axial length (AL) = 1.000; anterior chamber depth (ACD) = 0.997; IOL thickness (IOL LT) = 0.997; central corneal thickness (CCT) = 0.987). The mean AL measurement difference was 0.003 ± 0.021 mm (the 95% LoA ranged from −0.04 to 0.05); the mean ACD difference was −0.009 ± 0.025 mm (95% LoA, −0.06 to 0.04); mean LT difference was 0.001 ± 0.021 mm (95% LoA, −0.04 to 0.04); and mean CCT difference was 1.4 ± 5.4 μm (95% LoA, −9 to 12).

**Conclusion:**

The study shows small, non‐significant differences between the biometric measurements obtained with REVO NX SW‐OCT and IOLMaster 700 SS‐OCT in pseudophakic eyes. However, SW‐OCT offered significantly lower ACD and LT measurement failure rates. With high‐resolution imaging, SW‐OCT enables accurate assessment of IOL position relative to the posterior capsule and visualization of capsular fibrosis.

## Introduction

Split‐Window optical coherence tomography OCT (SW‐OCT) biometry is a new imaging technique that allows conventional anterior/posterior segment OCT devices to obtain ocular axial measurements by shifting the scanning window position along the measurement axis (Sikorski & Suchon 2019). Our previous study showed that biometric measurements obtained with SW‐OCT implemented in a commercially available spectral domain (SD) OCT device like REVO NX (Optopol Technology, Zawiercie, Poland) in healthy and cataractous eyes were comparable to those obtained using the swept‐source (SS) OCT‐based optical biometer IOLMaster 700 (Carl Zeiss Meditec AG, Jena, Germany) (Sikorski & Suchon 2019). Thus, the REVO NX has added new functionality to the potential of posterior segment OCT devices The high resolution of the REVO NX (typically seen in retinal imaging devices) exceeds that of the IOLMaster 700 over four‐fold and provides both biometric measurements and accurate information about the central macular structure. It also enables fine and accurate manual border correction of the measured ocular structures, which makes it easier to ascertain ocular biometry even in especially difficult cases.

The purpose of this study is to evaluate the utility of SW‐OCT in assessing ocular axial dimensions as well as imaging the intraocular lens (IOL) structure and posterior lens capsule in pseudophakic eyes.

## Material and methods

### Subjects

Sixty‐nine eyes of 69 patients, who underwent cataract surgery with IOL implantation, were assessed. The mean time from cataract surgery was 3.1 ± 1.2 months. A comprehensive ocular assessment, including subjective refraction, non‐contact tonometry as well as a slit‐lamp and fundus examination, was carried out for all eyes. The SW‐OCT (REVO NX, λ = 840 nm) non‐mydriatic measurements of 52 eyes (mean age 65.2 ± 13.8 years; 27 females) were compared with biometry values obtained using SS‐OCT (IOLMaster 700, λ = 1050 nm). If a measurement of an individual parameter was not feasible for either instrument after two attempts, a measurement failure was recorded for this parameter and device. Furthermore, an additional 17 eyes with more complex anatomy (*e.g*. piggyback and phakic lenses) were examined to determine the utility of the SW‐OCT in challenging cases. The study protocol was in accordance with the Declaration of Helsinki, and the Institutional Ethics Committee approval was obtained. All participants signed an informed consent.

### Measurements and analysis

In order to obtain SW‐OCT measurements the REVO NX, the anterior and posterior segments OCT device, was used (software ver. 9.0.0). We presented a detailed description and the theoretical assumptions of SW‐OCT biometry in our previous article, which introduced the technique (Sikorski & Suchon 2019). Briefly, SW‐OCT records the following structures located along the optical axis of the eye: the anterior and posterior boundary of the cornea, the anterior and posterior boundaries of the lens, as well as the posterior boundary of the retina. As the conventional OCT systems for posterior and anterior segment imaging cannot visualize the entire ocular axial structure on a single scan, the REVO NX relies on an accurate identification of measured structures and their individual acquisition.

The measurements are performed in four measurement windows, sized 3.0 mm × 2.5 mm each. These cover different ocular structures: the anterior and posterior boundary of the cornea (the first window), the anterior boundary of the crystalline lens or IOL (the second window), the posterior boundary of the crystalline lens (the third window) and the central retina (the fourth window). Subsequent cross‐sectional scans are obtained in consecutive measurement windows as the imaging window is shifted along the Z‐axis by means of the C‐gate shift along the measurement axis. Translucent IOLs are seen in SW‐OCT cross‐sectional scans as two curved lines corresponding to their anterior and posterior boundaries. As the IOL thickness is smaller than the width of the 2nd and 3rd measurement window (2.5 mm), the IOL will be visualized in both windows. Its anterior and posterior boundaries will be determined within the 2nd and 3rd measurement windows, respectively. The 3rd measurement window also acquires the posterior capsule, the anterior vitreous surface and the spatial relationship between the IOL and the posterior capsule.

Each SW‐OCT measurement taken as a part of this study consisted of 10 repetitions. At the end of the measurement, the mean value and SD were calculated automatically for axial length (AL), anterior chamber depth (ACD, as measured from the corneal epithelium to the lens), IOL thickness (LT) and central corneal thickness (CCT). The measurement and boundary identifications were fully automated, although manual correction by the clinician was also possible if deemed necessary. The AL measurements obtained using SS‐OCT were the mean values of three scans in each of six meridians. The scans were checked for foveal position to ensure the correct axial alignment and the measurement was repeated if the patient had not correctly fixated. The quality control criteria were applied for IOLMaster 700 as per manufacturer recommendations.

### Statistical analysis

Statistical analysis was performed using Statistica 13.1 (Dell Inc., USA). The size of the sample was calculated to be 30 eyes on the basis of the equivalence range (based on the pilot study). The range was established with consideration of the accuracy of the measurement readings of the devices and the level at which differences are clinically insignificant. It was assumed that mean differences between measurements should not exceed ±0.01 mm for the AL, ACD and LT, and ±5 μm for the CCT measurement. According to the recommendations on the estimation of the size of samples, the level of type I error was set at 0.05 and the power was assumed at 0.8.

The normality of data was assessed with the Shapiro–Wilk method. All parameters were distributed normally. The assumptions for using parametric tests for dependent variables were also evaluated. The distribution of differences was normal for all parameters. To assess agreement between the SW‐OCT and the SS‐OCT biometry for AL, ACD, LT and CCT, paired samples *t*‐test and Bland–Altman plots were performed and 95% limits of agreement (LoA) were calculated by the mean difference ± 1.96 SD (Bland & Altman 1986). A p‐value less than 0.05 was considered significant.

## Results

The range of values of ocular axial dimensions measured using the SW‐OCT were: AL = 19.66–27.31 mm, ACD = 3.61–6.12 mm, IOL LT = 0.55 –1.51 mm and CCT = 0.490–0.730 mm. The ACD and LT measurements were corrected manually in 9 and 11 eyes, respectively. Axial length (AL) measurements proved impossible to perform accurately in one eye using both SW‐OCT and SS‐OCT. The measurement failure rate with SW‐OCT was 1 eye for ACD, 2 eyes for LT and 0 eyes for CCT. The failure rate when measuring ACD, LT and CCT with SS‐OCT was 22, 30 and 2 eyes, respectively.

**Fig. 1:**
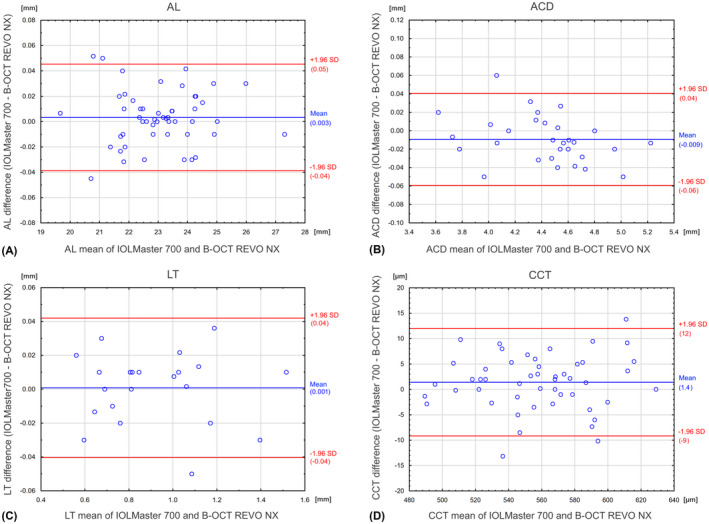
Bland–Altman plots for AL (A) ACD (B), LT (C) and CCT (D). The red lines indicate the 95% limits of agreement.

Bland–Altman plots for comparisons between SS‐OCT and SW‐OCT values for AL, ACD, LT and CCT are presented in Fig. 1. Table 1 shows a comparison of these values. For those eyes that could be measured by both biometers, the mean AL measurement difference was 0.003 ± 0.021 mm (the 95% LoA ranged from −0.04 to 0.05); the mean ACD difference was −0.009 ± 0.025 mm (95% LoA, −0.06 to 0.04); the mean LT difference was 0.001 ± 0.021 mm (95% LoA, −0.04 to 0.04); and the mean CCT difference was 1.4 ± 5.4 μm (95% LoA, −9 to 12).

**Table 1:** Comparison of measurements between REVO NX and IOLMaster 700.

	*n*	Device	Mean	SD	Paired samples *t*‐test (p‐value)	Range	Difference of the means*	SD for difference of the means	95% CI for differences of the means	ICC
Intraocular lenses										
Axial length (mm)	51	REVO NX	23.03	1.40	0.2772	19.66–27.31	0.003	0.021	−0.003 to 0.009	1.000
		IOLMaster 700	23.04	1.40		19.67–27.30				
Anterior Chamber depth (mm)	30	REVO NX	4.43	0.38	0.0533	3.61–5.23	−0.009	0.025	−0.019 to 0.000	0.997
		IOLMaster 700	4.42	0.37		3.63–5.22				
Lens thickness (mm)	22	REVO NX	0.92	0.26	0.8660	0.55–1.51	0.001	0.021	−0.009 to 0.010	0.997
		IOLMaster 700	0.92	0.26		0.57–1.52				
Central Corneal thickness (μm)	50	REVO NX	557	34	0.0721	490.0–629.0	1.4	5.4	−0.1 to 2.9	0.987
		IOLMaster 700	559	35		488.7–629.0				

ICC = intraclass correlation coefficient.

* Difference of the means was computed by subtracting REVO NX values form IOLMaster 700 values.

**Fig. 2:**
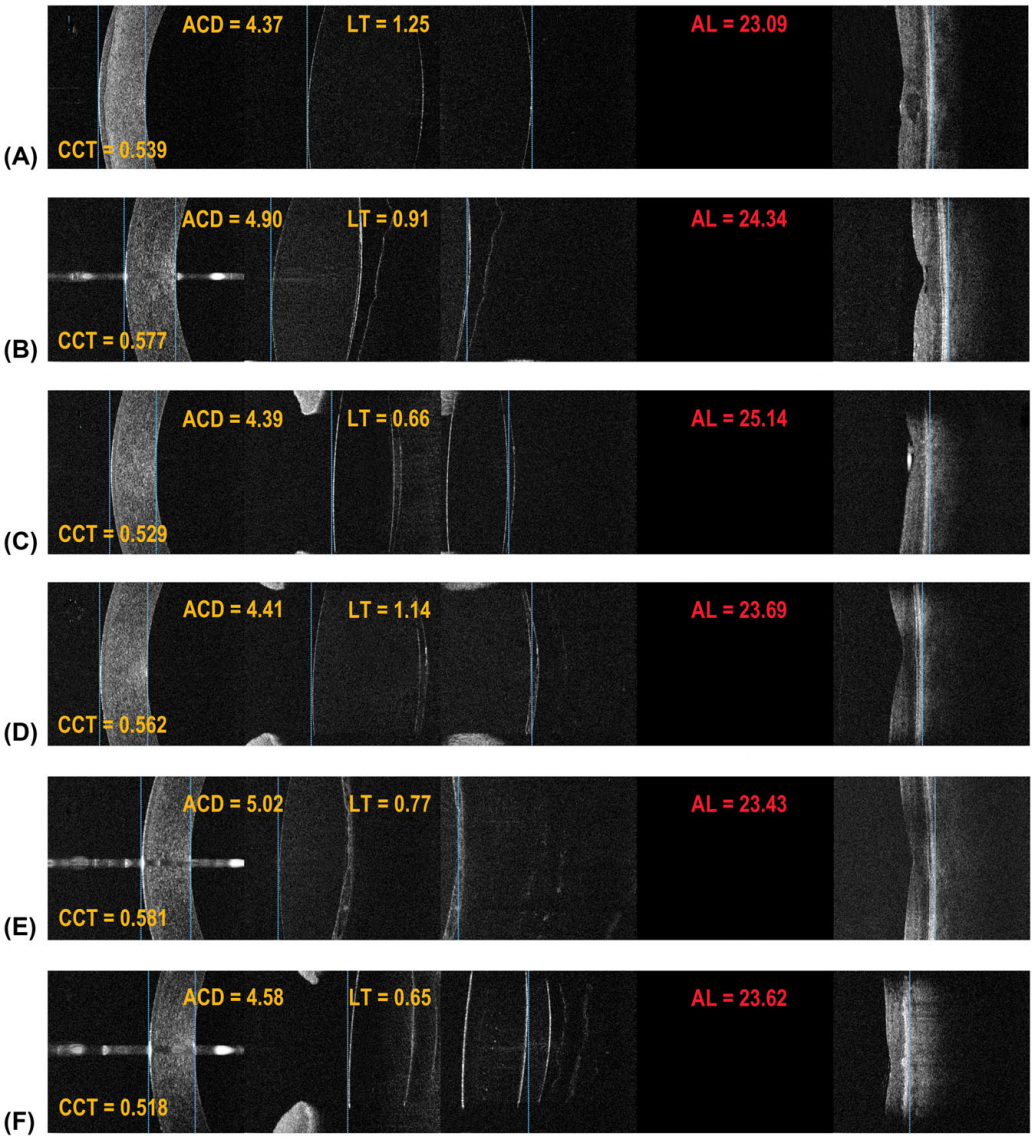
Split‐Window OCT (SW‐OCT) in eyes with IOLs. (A) The IOL properly situated within the posterior capsule, high‐resolution imaging enables accurate assessment of both corneal (keratopathy) and macular (macular oedema) pathologies; (B) A minimum IOL‐posterior capsule distance with visible anterior vitreous detachment; (C) A visible space between the posterior IOL surface and the posterior capsule, high‐resolution imaging enables visualizing the posterior surface of silicone oil tamponade in the prefoveal area, which was not visualized using the SS‐OCT; (D) A significant IOL‐posterior capsule distance; there is a central hyper‐reflective area on the capsule, which corresponds to early opacity; (E) A posterior capsular opacity casting a small shadow onto the retina; (F) A large IOL‐ posterior capsule distance with clearly visible vitreous surface.

**Fig. 3:**
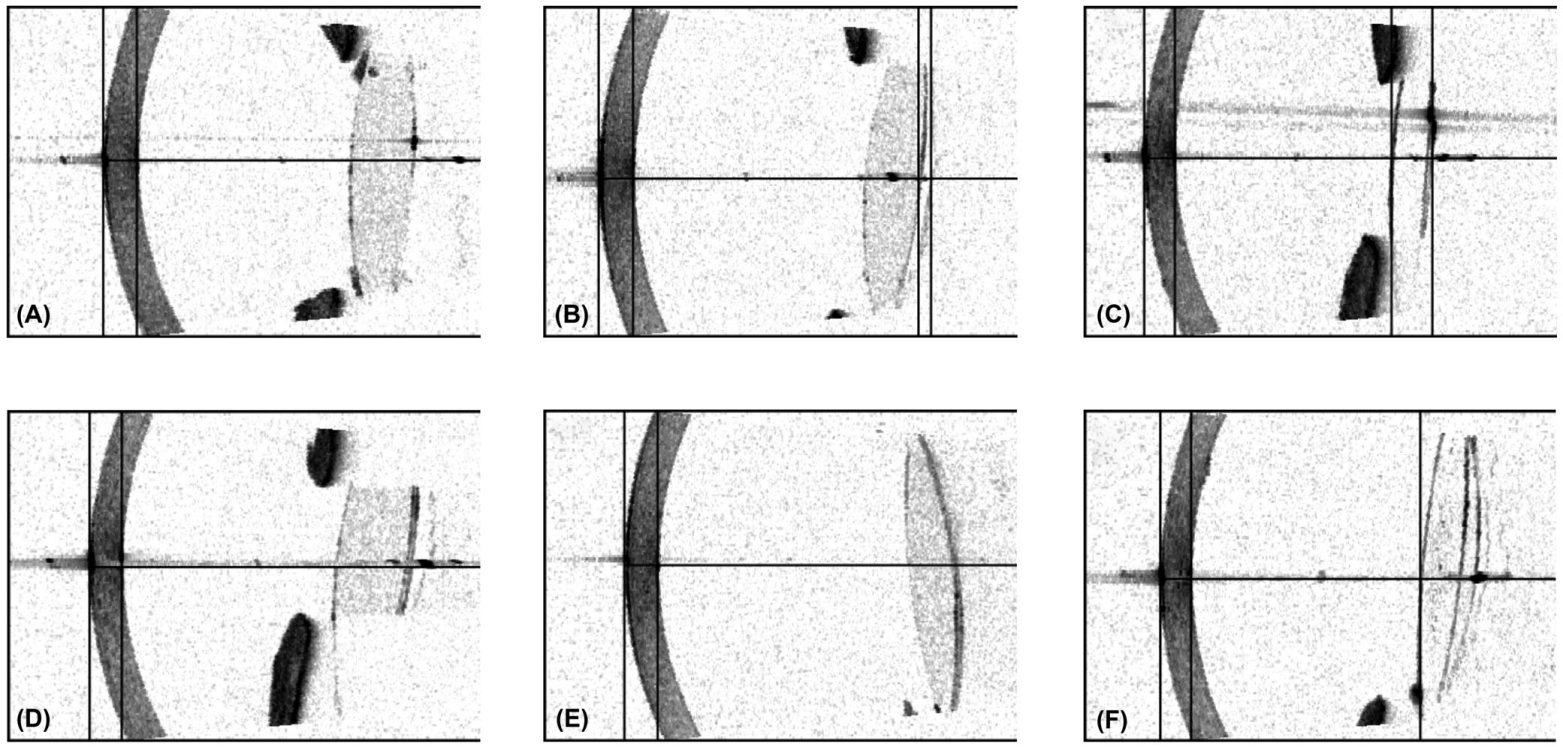
Corneal and IOL scans of eyes from Fig. 2, acquired using the SS‐OCT. (A) IOL boundaries are not identified, insufficient resolution makes it impossible to assess the cornea, which has a stromal irregularity clearly visible in SW‐OCT; (B) anterior vitreous surface is mistakenly identified as posterior IOL surface, the posterior IOL surface‐posterior capsule distance is not visible due to the lower resolution of the SS‐OCT; (C) IOL thickness measurement does not account for the lens posterior capsule distance seen in Fig. 2C; (D) IOL boundary identification failure, a significant IOL‐posterior capsule distance seen using the SW‐OCT is not visualized with the SS‐OCT, it is mistakenly presented as a posterior capsule opacification; (E) IOL boundary identification failure, posterior capsular opacity cannot be visualized precisely using the SS‐OCT and only presents as a non‐specific hyperreflectivity along the posterior surface of the IOL; (F) SS‐OCT posterior IOL boundary identification failure, clearly visible IOL‐posterior capsule distance.

**Fig. 4:**
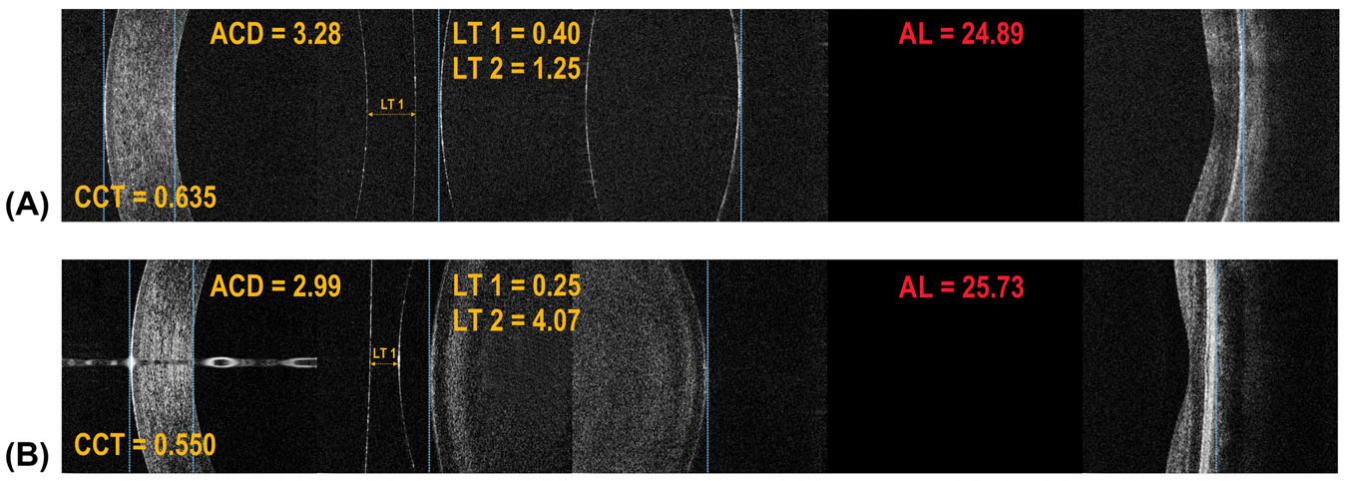
Split‐Window OCT (SW‐OCT) in eyes with special IOLs. (A) Two intraocular lenses in one eye (piggyback lens). Notably, it is possible to ascertain the relative position of both IOLs and their profile. (B) A phakic IOL and the crystalline lens in an eye, high‐resolution imaging makes it possible to accurately determine IOL location.

Figure 2 presents examples of SW‐OCT scans of pseudophakic eyes with varying IOL‐posterior capsule distances. Corresponding cross‐sections of the cornea, anterior chamber and IOL obtained using the SS‐OCT are shown in Fig. 3. The SW‐OCT scans in eyes with special IOLs (piggyback and pseudophakic lenses) are shown in Fig. 4.

## Discussion

The SW‐OCT offers advantages over conventional ocular biometry combined with those of anterior and posterior segment OCT. As a result, it is possible to measure the ocular axial dimensions whilst maintaining high‐resolution imaging, which enables acquisition of high‐quality corneal, lens and retinal cross‐sectional scans (Sikorski & Suchon 2019) (Wylęgała et al. 2020). Therefore, not only the AL and CCT, but also ACD and IOL LT can be obtained with high accuracy in pseudophakic eyes. Additionally, the IOL position relative to the posterior capsule as well as the condition of the posterior capsule itself can be ascertained during a single assessment. It is worth pointing out here that the transverse resolution of the REVO NX in each of the four measurement windows obviously differs due to the change in the magnification of the optical system. In the case of an ideal eye, it is equal to 27 μm for the cornea and 19 μm for the retina. The measured value of the nominal axial resolution is 5 μm in air, but this is defined for a single reflecting surface such as a mirror in air. For the scattering surfaces considered in these analyses, the accuracy of the thickness determination will depend on many other factors including speckle size and individual variable reflectance and dispersion parameters. Hence, the precision resulting from statistical analysis in actual measurements may be lower than the nominal axial resolution.

**Fig. 5:**
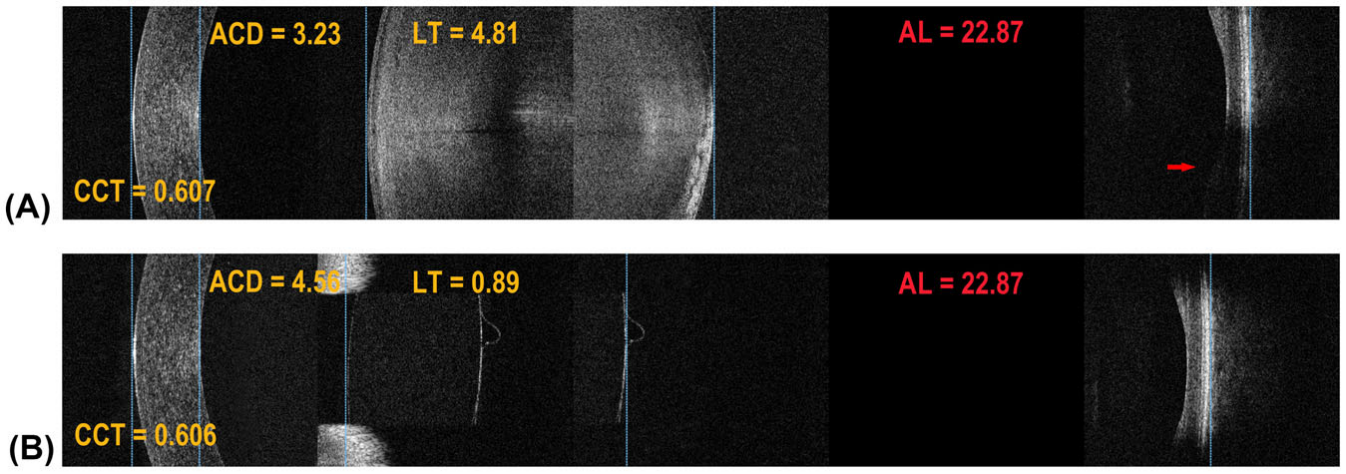
Split‐Window OCT (SW‐OCT) before and after cataract surgery (A) preoperative measurements. There is an evident posterior subcapsular opacity casting a shade onto the retina (red arrow); (B) postoperative measurements with the IOL visible in place. As the pupil is narrow, the iris casts a shade onto the retina. The posterior capsular fold is visible.

Our work shows that high‐resolution imaging is necessary to properly assess an IOL position relative to the posterior capsule, capsular fibrosis and to obtain the distance between the IOL and the posterior capsule. For example, even with a lens capsule distance above the minimum, the SS‐OCT is not able to visualize it (Fig. 2). Dedicated anterior segment OCT devices can be used to accurately visualize the anterior segment (Han et al. 2016; Ang et al. 2018; Venkateswaran et al. 2018). One such platform which offers an excellent visual analysis of the entire anterior segment with the additional ability to measure the ocular AL is the Anterion (Heidelberg Engineering GmbH, Heidelberg, Germany) (Ruiz‐Mesa et al. 2020). However, it does not provide information about the retina and does not visualize the posterior pole at the site where the biometric measurements are being collected. Therefore, a separate OCT device is needed to assess the retinal structure and its effect on vision. Even biometers that provide OCT images of the complete longitudinal section of the eye, such as the IOL Master 700 and Argos (Movu Inc., Santa Clara, CA) cannot replace a macular OCT device due to significant deficiencies in their retinal scanning quality (Hirnschall et al. 2016; Sikorski and Suchon 2019; Omoto et al. 2019; Tognetto et al. 2019). In this context, the REVO NX can be considered a multimodal imaging platform optimized for the posterior segment, which provides accurate measurements of ocular axial dimensions, imaging of the anterior segment structures and does not require an additional device to assess the retina. A high‐resolution central retinal scan is an integral part of the assessment. It is a feature unique to the SW‐OCT, which further facilitates accurate assessment of patient fixation during measurement. Hence, the SW‐OCT can be routinely used after cataract surgery for a comprehensive assessment of ocular structures along the optic axis (Fig. 5). If a central retinal pathology is suspected to be the cause of suboptimal postoperative vision, it can be confirmed using the SW‐OCT. By manually setting the boundary markers, optical axial distances can be measured. Such measurements are made in close proximity to the optical axis of the eye, so we used the average refractive index to calculate geometric distances and used simplified raytracing to account for corneal curvature. This approach is particularly useful in more complex cases, such as piggyback and pseudophakic IOLs (Fig. 4), and in patients with retinal structure abnormalities (Sikorski and Suchon 2019). Other SS‐OCT biometers do not offer such functionality. At this point, it should be noted that it is possible to make corrections to AL measurements on morphological images of ocular tissues. In the case of other optical biometers, for example, IOLMaster 500 (Carl Zeiss AG, Oberkochen, Germany), Lenstar 900 (Haag‐Streit AG, Koeniz, Switzerland), Pentacam AXL (Oculus, Wetzlar, Germany), Galilei G6 (Ziemer, Port, Switzerland), OA‐2000 (Tomey Corporation, Nagoya, Japan), Argos (Movu Inc., Santa Clara, CA) AL measurements rely only on optical echograms (where the intensity of the A‐scan is expressed by its height).

We have recently become aware that the ACD (epithelium to lens) measurement of the IOLMaster 700 was specifically set to match that of the original IOLMaster 500 rather than giving a true reading (Carl Zeiss, personal communication). It has been shown in many previous studies that the IOLMaster 500 always measures a lower ACD reading by an average of 0.21 mm (0.13–0.27) than all optical biometers from other manufacturers so far compared with it (Hoffer et al. 2010; Hoffer & Savini 2016; Shammas et al. 2016; Hoffer et al. 2016a; Hoffer et al. 2016b). However, the ACD measurements with IOLMaster 500 were found to be highly correlated with IOLMaster 700 (Akman et al. 2016). Since this study shows that the REVO NX SW‐OCT gives the ACD reading similar to the IOLMaster 700, we believe that both devices should review this so as to ensure reporting the actual reading they obtain.

Some limitations of our study were a relatively small number of compared measurements, which is especially true for IOL LT and ACD, due to a high failure rate by SS‐OCT to properly identify their boundaries. A larger sample size may be a way to solve this difficulty in future studies. It should be noted that in our study, we measured and identified ocular structures using the REVO NX SW‐OCT commercial software, which was not optimized for IOL boundary detection. Its modification will increase the rate of fully automated measurements.

Split‐Window OCT (SW‐OCT) biometry is a novel technique for measuring ocular axial dimensions that can be potentially implemented into both SD and SS‐based anterior/posterior segment OCT devices. It can be used not only in eyes with crystalline lenses, but also those after IOL implantation, including phakic IOL eyes and piggyback lenses. High‐resolution imaging makes it ideal for assessing patient eligibility for cataract surgery whilst providing information about the central retinal structure, as well as for assessing eyes after cataract surgery. This also makes it a useful tool for future research.
